# Long-term, telephone-based follow-up after stroke and TIA improves risk factors: 36-month results from the randomized controlled NAILED stroke risk factor trial

**DOI:** 10.1186/s12883-018-1158-5

**Published:** 2018-09-21

**Authors:** Joachim Ögren, Anna-Lotta Irewall, Lars Söderström, Thomas Mooe

**Affiliations:** 1 Department of Public Health and Clinical Medicine, Umeå University, Östersund, Sweden; 2 Unit of Research, Development and Education, Östersund, Sweden

**Keywords:** Stroke, TIA, Secondary prevention, Modifiable risk factors, Blood pressure, Cholesterol, Randomized controlled study, Telemedicine, Nurses

## Abstract

**Background:**

Strategies are needed to improve adherence to the blood pressure (BP) and low-density lipoprotein cholesterol (LDL-C) level recommendations after stroke and transient ischemic attack (TIA). We investigated whether nurse-led, telephone-based follow-up that included medication titration was more efficient than usual care in improving BP and LDL-C levels 36 months after discharge following stroke or TIA.

**Methods:**

All patients admitted for stroke or TIA at Östersund hospital that could participate in the telephone-based follow-up were considered eligible. Participants were randomized to either nurse-led, telephone-based follow-up (intervention) or usual care (control). BP and LDL-C were measured one month after discharge and yearly thereafter. Intervention group patients who did not meet the target values received additional follow-up, including lifestyle counselling and medication titration, to reach their treatment goals (BP < 140/90 mmHg, LDL-C < 2.5 mmol/L). The primary outcome was the systolic BP level 36 months after discharge.

**Results:**

Out of 871 randomized patients, 660 completed the 36-month follow-up. The mean systolic and diastolic BP values in the intervention group were 128.1 mmHg (95% CI 125.8–130.5) and 75.3 mmHg (95% CI 73.8–76.9), respectively. This was 6.1 mmHg (95% CI 3.6–8.6, *p* < 0.001) and 3.4 mmHg (95% CI 1.8–5.1, *p* < 0.001) lower than in the control group. The mean LDL-C level was 2.2 mmol/L in the intervention group, which was 0.3 mmol/L (95% CI 0.2–0.5, *p* < 0.001) lower than in controls. A larger proportion of the intervention group reached the treatment goal for BP (systolic: 79.4% vs. 55.3%, *p* < 0.001; diastolic: 90.3% vs. 77.9%, *p* < 0.001) as well as for LDL-C (69.3% vs. 48.9%, *p* < 0.001).

**Conclusions:**

Compared with usual care, a nurse-led telephone-based intervention that included medication titration after stroke or TIA improved BP and LDL-C levels and increased the proportion of patients that reached the treatment target 36 months after discharge.

**Trial registration:**

ISRCTN Registry ISRCTN23868518 (retrospectively registered, June 19, 2012).

**Electronic supplementary material:**

The online version of this article (10.1186/s12883-018-1158-5) contains supplementary material, which is available to authorized users.

## Background

Stroke is a major cause of mortality and morbidity worldwide. Today, more patients than ever survive strokes, thereby increasing the prevalence of stroke survivors [1]. These patients have an increased risk of new vascular events [2–4], but this risk can be reduced by hypertension treatment as well as by statin treatment [5, 6]. Current guidelines therefore recommend both [7, 8].

Notably, observational studies show that after stroke, only 25 to 49% of all patients reach treatment targets for blood pressure (BP) and only 14 to 77% reach treatment targets for low-density lipoprotein cholesterol (LDL-C) [9–13]. Strategies to improve patient control of modifiable risk factors have been tested in randomized controlled trials (RCTs) with heterogeneous results [13–18]. Considering the high prevalence of stroke survivors and the limited available resources for public health care, involving health care professionals other than physicians as well as telemedicine-based strategies might offer alternative cost-effective solutions to improve secondary prevention.

The RCTs of risk factor interventions delivered mainly by nurses or pharmacists have a variety of designs and have shown variable results [13, 14, 17, 19]. Most programs that improve risk factor control after stroke or transient ischemic attack (TIA) include medical treatments that can be adjusted by a physician or by a nurse or pharmacist [15, 17, 20, 21]. In a recent systematic review of telemedicine strategies in patients after a stroke or TIA, a meta-analysis including four studies showed significant improvements in BP [22] using telemedicine compared to usual care. The studies were heterogeneous in terms of their methods and results, and the components, duration, and intensity of the follow-up programs also varied in the studies. However, in two of the four studies, the intervention program included follow-up and adjustment of medical treatment.

The nurse-based age independent intervention to limit evolution of disease after stroke or TIA (NAILED) trial combined a nurse-based, telemedicine strategy with systematic review of medical treatment, including titration of medicine. This approach decreased BP and LDL-C compared significantly better than in a control group at 12 months [20]. However, the secondary preventive perspective is generally considerably longer in terms of treatment duration, and there is currently insufficient knowledge of how to perform cost-effective follow-up to ensure long-term adherence to risk factor treatment goals. Currently, the only available study with an intervention that continued beyond 12 months after stroke or TIA did not show significant improvements in BP or LDL-C [23], but it did not use medication titration

### Objectives

The primary aim of the present study was to investigate whether the NAILED trial intervention improved BP values and LDL-C levels 36 months after stroke or TIA compared to usual care. The secondary aim was to evaluate whether a larger proportion of the intervention group reached set treatment targets. Finally, we aimed to investigate whether there were any trends in the effects of the intervention during the study period. An abstract of the current study has been previously published [24].

## Methods

### Trial design

The NAILED stroke risk factor trial was a population-based RCT with two parallel groups and an allocation ratio of 1:1. The design of the study was described previously in the published study protocol [25] and has been used and described in the published analysis of the 12 months follow-up of the NAILED-stroke trial [20]. It is described briefly below.

### Participants

All patients treated with an intracerebral hematoma (ICH), ischemic stroke (IS) or TIA at Östersund hospital, the only hospital in the county of Jämtland Sweden between January 1, 2010 and December 31, 2013 were considered for participation in the study. To be considered eligible the participants also had to be able to participate in a telephone-based follow-up and sign an informed, written consent. Patients with aphasia, impaired hearing, cognitive impairment, or severe, often terminal disease, were excluded..

### Randomization and blinding

Eligible patients were randomly assigned to the intervention group or the control group. The randomized allocation sequence was computer-generated in blocks of four and stratified for sex and for degree of disability (modified Rankin Scale **<** 3 or ≥ 3). The resulting group allocation was not blinded to participants, the study team, or other caregivers.

### Data collection and follow-up

Measurments of BP and blood lipids were performed at the patients’ closest healthcare facility at 1, 12, 24, and 36 months post-discharge. A study nurse then interviewed participants in both the intervention and control groups about their compliance with recommended treatment, sense of well-being, use of tobacco, and physical activity level. The information was collected systematically according to the variables in the study protocol. To perform the follow-up a nurse working 0.5–0.75 of a full time was required. The study nurse was experienced in stroke care and had participated in courses in motivational interviewing (MI) and good clinical practice (GCP).

### Intervention

**Fig. 1:**
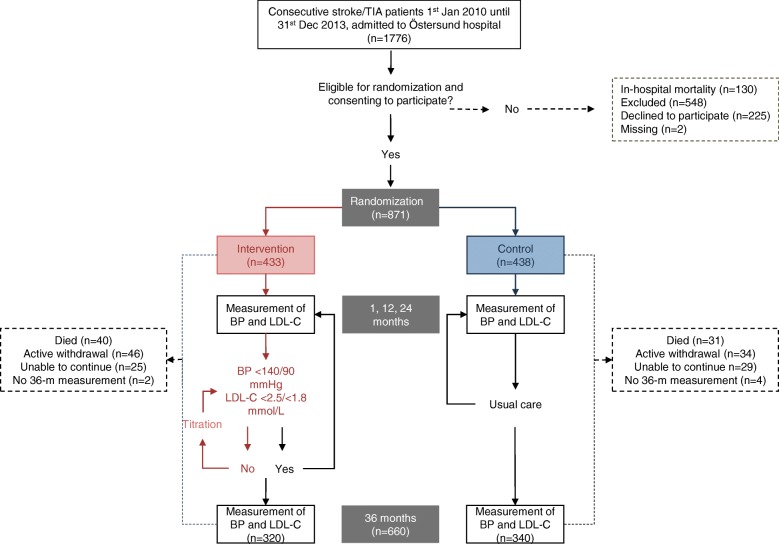
Study flow chart. *TIA* transient ischemic attack, *BP* blood pressure, LDL-C low-density lipoprotein cholesterol

The intervention group received telephone-based counselling and an assessment of their pharmacological treatment [25]. A study physician was consulted to assess and adjust the medical treatment when the participants did not achieve the set target for LDL-C and/or BP. There were no pre-specified algorithm to the pharmacological adjustments; rather, individualized for each participant. The process was repeated after approximately 4 weeks, when necessary (see flow chart in Fig. 1). Lipid-lowering therapy was restricted to patients with ischemic events.

In the control group, treatment was generally initiated in-hospital and after discharge they received secondary preventive care according to local standards, most often by each patient’s general practitioner.

### Outcomes

Outcome variables were measured at 1, 12, 24 and 36 moths and included sitting systolic blood pressure (SBP) and diastolic blood pressure (DBP), LDL-C, and the proportion of patients reaching set targets for these variables. Sitting SBP at 36 months was analyzed as the primary outcome, and the other variables were analyzed as secondary outcomes. SBP < 140 mmHg, DBP < 90 mmHg, and an LDL-C value < 2.5 mmol/L (or < 1.8 mmol/L in patients with diabetes) were considered to be within the target range according to local guidelines at the time of the assessments.

BP was measured once in the seated position after 5 min of rest. LDL-C values were calculated using the Friedewald formula. Cause of death data were obtained from the national cause of death register.

### Sample size

To reliably detect a difference of 5 mmHg between the groups for the mean SBP, we needed study groups of 180 participants (standard deviation 19, mean SBP 140 versus 135, alpha 0.05 two-tailed, power 80%). Study groups of at least 200 participants were planned to allow for drop-outs. This sample size was also adequate for detection of a group difference of 0.3 mmol/L in LDL values.

### Statistical methods

The analyses were performed according to the intention to treat principle. Baseline characteristics comparisons between groups were performed using an independent sample t-test or a chi-square test as appropriate.

We calculated the adjusted mean differences between groups (intervention vs. control) in BP and LDL-C levels at 36 months using a general linear model adjusted for sex and degree of disability in order to reflect the stratified randomization. We used paired sample t-tests to evaluate changes in mean BP and LDL-C values between 1 and 36 months within a single group.

All analyses were performed using SPSS software, version 24.0, and we defined the significance threshold at the level of *p* = 0.05.

### Trial registration

The NAILED stroke risk factor trial is registered in the ISRCTN registry (ISRCTN23868518). The ICMJE strict requirement of prospective registration of clinical trials came to our attention when the recruitment had already begun. The study was therefore retrospectively registered on June 19, 2012. The authors confirm that all ongoing and related trials for this intervention are now registered.

## Results

Out of the 871 randomized patients, 660 participants completed the 36-month follow-up and were included in the analysis (mean age: 69.6 years, 40.8% women, 58.6% with IS, 3.5% with ICH, and 37.9% with TIA). Figure 1 shows the flow chart of the participants. The baseline characteristics of the participants that are included in the final analysis were well balanced, except for diabetes, which was more common in the control group. Table 1 shows the baseline data of the participants in the final analysis, and Additional file 1: Table S1 shows the baseline characteristics of all of the participants who were randomized in the study.

**Table 1:** Baseline characteristics of the study participants

	Intervention group (*n* = 320)	Control group (*n* = 340)	*P* value
Mean age, years	69.9	69.3	ns
Women no. (%)	130 (40.6)	139 (40.9)	ns
Qualifying event no. (%)			
Ischemic stroke	181 (56.6)	206 (60.6)	ns
Intracerebral hematoma	11 (3.4)	12 (3.5)	ns
TIA	128 (40.0)	122 (35.9)	ns
mRS 3–5 no. (%)	37 (11.6)	31 (9.1)	ns
Medical history no. (%)			
Stroke	41 (12.9)	37 (10.9)	ns
Myocardial infarction	22 (6.9)	30 (8.8)	ns
Heart failure	8 (2.5)	9 (2.6)	ns
Atrial fibrillation	47 (15.2)	52 (15.4)	ns
Diabetes	45 (14.1)	68 (20.1)	0.049
Smoker no. (%)	42 (13.1)	48 (14.1)	ns
Medications at 1 month no. (%)			
Antihypertensive drug	231 (72.2)	264 (77.6)	ns
Statin	253 (79.8)	276 (81.7)	ns
Antiplatelet drug	253 (79.1)	276 (81.2)	ns
Anticoagulant drug	48 (15.1)	45 (13.3)	ns
Baseline values ± SD			
SBP (mmHg)	136.9 ± 16.7	137.2 ± 18.5	ns
DBP (mmHg)	80.8 ± 11.6	80.2 ± 10.4	ns
LDL-C (mmol/L)	2.5 ± 0.8	2.4 ± 0.8	ns

*mRS* modified Rankin scale, *TIA* transient ischemic attack, *SBP systoilc blood pressure, DBP diastolic blood pressure, LDL-C* low-density lipoprotein cholesterol

Of the 211 participants that did not complete the 36-month follow-up, 80 patients chose to discontinue follow-up, 54 were not able to continue due to severe disease, and 71 died. Six participants wanted to continue to participate in the study but were unable to complete the 36-month follow-up. The participants who did not complete the 36-month follow-up were older and had more co-morbidities. A total of 99 patients died between randomization and the 36-month follow-up; of these, 21 of 55 deaths in the intervention group and 17 of 44 deaths in the control group were classified as cardiovascular-related deaths. The intervention group and the control group did not differ significantly in terms of the proportions of cardiovascular or all-cause mortality (*p* = 0.51 and *p* = 0.24).

### SBP at the 36-month follow-up

At 36 months, the mean adjusted SBP was 128.1 mmHg (95% CI 125.8–130.5) in the intervention group and 134.2 mmHg (95% CI 131.8–136.6) in the control group, with a difference of 6.1 mmHg (95% CI 3.6–8.6, *p* < 0.001) between the groups. The decreases in BP compared to the 1 month measurements were 8.1 mmHg (95% CI 5.8–10.3) and 2.3 mmHg (95% CI 0.1–4.4) in the intervention and control groups, respectively.

### DBP at the 36-month follow-up

The mean adjusted DBP values in the intervention and control groups were 75.3 mmHg (95% CI 73.8–76.9) and 78.8 mmHg (95% CI 77.2–80.3), respectively, with a difference of 3.4 mmHg (95% CI 1.8–5.1, *p* < 0.001) between the groups. The mean DBP decreased between 1 and 36 months by 4.4 mmHg (95% CI 2.9–5.8) and 0.2 mmHg (95% CI -1.0–1.5), respectively.

### LDL-C at the 36-month follow-up

The mean adjusted LDL-C values in the intervention and control groups were 2.2 mmol/L (95% CI 2.1–2.4, 86.5 mg/dL) and 2.5 mmol/L (95% CI 2.4–2.7, 98.1 mg/dL), respectively, a mean difference of 0.3 mmol/L (95% CI 0.2–0.5, *p* < 0.001). The decrease in the intervention group was 0.2 mmol/L (95% CI 0.1–0.3), while there was a significant increase of 0.1 mmol/L (95% CI 0.0–0.2) in the control group.

### Proportion of patients reaching the treatment targets

**Fig. 2:**
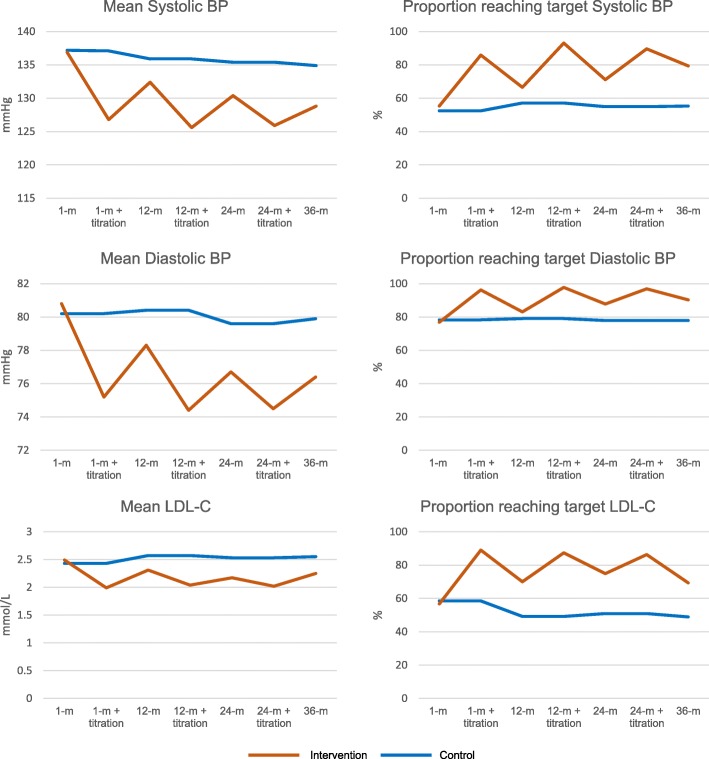
Unadjusted mean values and proportions of participants reaching targets for SBP, DBP, and LDL-C at 1, 12, and 24 months before and after medication titration and at 36 months. BP blood pressure, LDL-C low-density lipoprotein cholesterol, m months

At 36 months, 79.4% and 55.3% of participants reached the treatment target for SBP in the intervention and control groups, respectively (*p* < 0.001). The corresponding proportion were 90.3% vs. 77.9% (*p* < 0.001) for DBP and 69.3% vs. 48.9% (*p* < 0.001) for LDL-C. Figure 2 shows the results for 36 months as well as for 1, 12, and 24 months before and after titration of medication.

### Trends over time

**Fig. 3:**
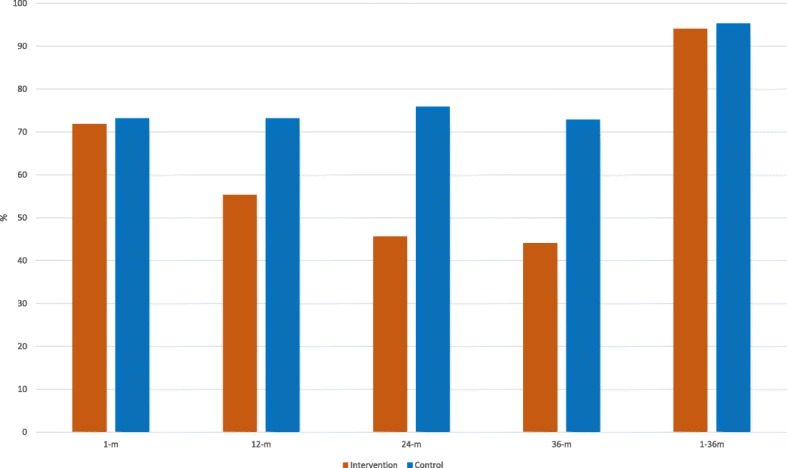
Proportion of participants with at least one SBP, DBP, or LDL-C measurement above the treatment target at 1, 12, 24 and 36 months follow up and during the entire study period. m months

At 1 month, 71.9% and 73.2% (*p* = 0.727) of the participants in the intervention and the control groups, respectively, had at least one LDL-C, SBP, or DBP measurement that did not reach the treatment target. At 36 months, the corresponding percentages were 44.1% and 72.9% (*p* < 0.001). Only 5.9% and 4.7% (*p* = 0.493) of the participants were below the treatment target values at all measurements during the study period (Fig. 3). The difference in the mean SBP and DBP values between the two groups increased during the study period, while the difference at 12 months remained unchanged at 36 months for LDL-C (Fig. 2).

## Discussion

The present study analyzed 660 participants at the end of 36-month follow-up. The results showed that nurse-led, telephone-based, secondary preventive follow-up that included medication titration improved SBP, DBP, and LDL-C 36 months after a stroke or TIA compared to the usual-care control group. Furthermore, a significantly larger proportion of participants in the intervention group reached the treatment targets for SBP and DBP as well as for LDL-C. The BP levels and the proportion of participants who reached the BP targets improved each year in the intervention group, but the proportion remained almost unchanged in the control group.

The final SBP and DBP levels were 6.1 mmHg and 3.4 mmHg lower in the intervention group than in the control group. These differences were comparable to the reductions seen in RCTs of antihypertensive treatment after stroke or TIA, which showed a 22 to 34% relative risk reduction of recurrent stroke [5, 26]. The LDL-C was reduced by 0.3 mmol/L in the intervention group, less than in the Stroke Prevention by Aggressive Reduction in Cholesterol Levels (SPARCL) study [6]. The clinical relevance of this reduction is uncertain.

Previous studies of interventions that aimed to improve the control of modifiable risk factors in the secondary prevention of stroke and TIA have had different designs and variable results [14–19, 21]. This makes it hard to distinguish which factors are responsible for observed benefits. A Cochrane meta-analysis from 2014 found a non-significant decrease in SBP and a non-significant increase in the proportion of patients who reached the treatment target for BP [14], but it did not identify any trends towards improvement in the LDL-C levels or in the proportion of participants reaching LDL-C treatment targets. Some studies have shown promising results for the improvement of modifiable risk factors after stroke or TIA and also in patients at risk of other cardiovascular diseases. These studies [15, 17, 19, 21, 27], as well as the present trial, all involved adjustment of pharmacological treatment by a nurse, a pharmacist, or a physician.

At discharge, 73% to 78% of the participants were on antihypertensive medication, and 80% were being treated with statins (Table 1). These proportions are comparable with the treatment data found in Riksstroke, the Swedish national stroke register [28]. Despite the high proportion of treated participants, only about half of the study participants reached the treatment target for SBP and LDL-C at the baseline assessment one month after discharge (Fig. 2). It is possible that the medication initiated during the hospital stay had not yet reached its full effect because there were plans to titrate it during follow-up or because the doses were inadequate.

During the follow-up of high-risk cardiovascular patients, failure to reach the treatment target should prompt a review of the medical treatment, an inquiry about adherence, and in most cases, reinforcement of medication to improve risk factor levels. In addition, the patient needs to be motivated and capable of following treatment recommendations to ensure adherence. In this study, the proportion of participants in the control group who reached treatment targets remained unchanged for BP and decreased for LDL-C over time, whereas the proportions increased in the intervention group. The lack of improvement was seen in the control group despite sending the follow-up measurements to each patient’s general practitioner. Plus, as a consequence of study participation, there was probably a higher-than-usual awareness of cardiovascular risk factors among the control group participants. The interference of the study with normal clinical practice in the control group was unavoidable. However, it might have been expected to contribute to an underestimation of the effect of the NAILED intervention. In addition, the control group achieved less than the intervention group despite a fairly equal number of healthcare contacts and risk factor assessments, at least during the first year, according to previously published results from the NAILED stroke risk factor trial [20]. We cannot know for sure why these contacts resulted in improved risk factor levels in the intervention group, but it is likely that the NAILED trial intervention, with systematic medication titration, decreased the risk that the physician would not respond to a value above the treatment target (i.e. therapeutic inertia), a problem that has been described in the follow-up after stroke or TIA [29]. Moreover, contact with the study nurse gave participants the chance to discuss their treatment and possible side effects, and this may have increased the participants’ adherence to medication. The combination of decreased therapeutic inertia and increased adherence might explain the positive results of the intervention.

### Time trends and the duration of follow-up

Stroke survivors have a life-long increased risk of recurrent stroke [3]. The effect of antihypertensive or lipid-lowering treatment on new vascular events in RCTs remains during follow-up periods of at least 36 months [5, 6, 30]. However, only two studies on interventions to improve secondary prevention after stroke or TIA had follow-up of more than 12 months [23, 31], and only one implemented interventions after 12 months [23]. None of the studies showed any significant improvements in BP or LDL-C levels. In the present study, we found a clinically relevant effect of the intervention on BP levels at 36 months. Furthermore, we found that the difference in BP between the study groups increased continuously during follow-up (Fig. 2). During the study period, the proportion of participants with at least one SBP, DBP, or LDL-C measurement that did not reach the target was nearly 95% in both the intervention and control groups (Fig. 3). However, the proportion of participants that needed treatment adjustment decreased continuously in the intervention group but not in the control group. In the intervention group, the proportion of participants that needed adjustment markedly increased between the end of each titration and the next follow-up (Fig. 2), and at 36 months, more than 40% of the participants in the intervention group had at least one SBP, DBP, or LDL-C value above the treatment target. Furthermore, almost all of the participants who did not need any medication adjustments at 1 month had at least one value above the treatment target during the follow-up period. Thus, regardless of the initial risk factor levels, life-long follow-up, similar to the routines for diabetic patients, seems necessary for an acceptable level of secondary prevention after stroke or TIA.

Continuous improvement was not seen for LDL-C, and the underlying reasons for this are unclear. This may be attributable to side effects and decreasing adherence to statin treatment. Further analyses are planned to explore this finding.

### Treatment target and endpoints

The present study focused on risk factor levels and on reaching treatment targets to decrease the risk of new vascular events [5, 6]. There are presently insufficient data about the optimal treatment target for BP after stroke or TIA, but guidelines recommend BP levels < 140/< 90 mmHg [7, 8]. Moreover, no trials have compared the use of different treatment targets for LDL-C after stroke or TIA. In this study, we chose BP levels < 140/< 90 mmHg and an LDL-C level < 2.5 mmol/L (< 1.8 in participants with diabetes) to be consistent with the treatment goals used in primary care during the study period, since the control group participants were treated in primary care.

At 36 months of follow-up, there were more deaths in the intervention group than in the control group (*n* = 55 vs. *n* = 44; *p* = 0.24). However, the present study was not designed and powered to investigate mortality or new vascular events. These analyses will be performed based on the entire follow-up period for the NAILED cohort.

### Strengths and limitations

With 871 participants randomized and 660 in the final analysis, this study is one of the largest in the field. We used a population-based approach and a simple follow-up routine in order to include a large proportion of the targeted population. Furthermore, the baseline treatments corresponded well with national level data in the Riksstroke registry. This should give the study high external validity. However, 35.7% of the patients could not participate in the study due to physical or cognitive impairment [32]. Moreover, 211 out of the 871 randomized participants did not reach the 3-year follow-up. This could be considered a weakness of the study, but it also reflects the reality of the characteristics of this study population. Severe co-morbidities and a high mortality rate in this population unavoidably increase the proportion of patients who cannot participate or who discontinue participation during follow-up. This limits the potential for secondary prevention.

## Conclusion

A nurse-led telephone-based intervention that included medication titration after stroke or TIA improved BP and LDL-C levels and increased the proportion of patients that reached treatment targets 36 months after discharge. The effect of the intervention on BP increased over time. If implemented, the NAILED strategy could improve BP and LDL-C levels in many stroke survivors.

## Additional file

Additional file1: **Table S1.** Baseline characteristics of the participants randomized in the trial. (DOCX 13 kb)

## Abbreviations

BP: Blood pressure
DBP: Diastolic blood pressure
ICH: Intracerebral hematoma
IS: Ischemic stroke
LDL-C: Low-density lipoprotein cholesterol
NAILED: Nurse based Age-independent Intervention to Limit Evolution of Disease after stroke or TIA
RCT: Randomized controlled trial
SBP: Systolic blood pressure
TIA: Transient ischemic attack

## References

[1] Feigin VL, Forouzanfar MH, Krishnamurthi R, Mensah GA, Connor M, Bennett DA, Moran AE, Sacco RL, Anderson L, Truelsen T (2014). Global and regional burden of stroke during 1990-2010: findings from the global burden of disease study 2010. Lancet.

[2] Feng WHR, Adams RJ (2010). Risk of recurrent stroke, myocardial infarction, or death in hospitalized stroke patients. Neurology.

[3] Mohan KMWC, Rudd AG, Heuschmann PU, Kolominsky-Rabas PL, Grieve AP (2011). Risk and cumulative risk of stroke recurrence: a systematic review and meta-analysis. Stroke.

[4] Touzé EVO, Chatellier G, Peyrard S, Rothwell PM, Mas JL (2005). Risk of myocardial infarction and vascular death after transient ischemic attack and ischemic stroke: a systematic review and meta-analysis. Stroke.

[5] Liu L, Wang Z, Gong L, Zhang Y, Thijs L, Staessen JA (2009). Blood pressure reduction for the secondary prevention of stroke: a chinese trial and a systematic review of the literature. Hypertens Res.

[6] Amarenco PBJ, Callahan A, Goldstein LB, Hennerici M, Rudolph AE, Sillesen H, Simunovic L, Szarek M, Welch KM, Zivin JA (2006). Stroke prevention by aggressive reduction in cholesterol levels (SPARCL) investigators. High-dose atorvastatin after stroke or transient ischemic attack NEJM.

[7] Kernan WN, Ovbiagele B, Black HR, Bravata DM, Chimowitz MI, Ezekowitz MD, Fang MC, Fisher M, Furie KL, Heck DV (2014). Guidelines for the prevention of stroke in patients with stroke and transient ischemic attack: a guideline for healthcare professionals from the american heart association/american stroke association. Stroke.

[8] Piepoli MF, Hoes AW, Agewall S, Albus C, Brotons C, Catapano AL, Cooney MT, Corra U, Cosyns B, Deaton C (2016). 2016 european guidelines on cardiovascular disease prevention in clinical practice: the sixth joint task force of the european society of cardiology and other societies on cardiovascular disease prevention in clinical practice (constituted by representatives of 10 societies and by invited experts)developed with the special contribution of the european association for cardiovascular prevention & rehabilitation (eacpr). Eur Heart J.

[9] Amar JCJ, Touzé E, Bongard V, Jullien G, Vahanian A, Coppé G, Mas JL (2004). ECLAT1 study investigators. Comparison of hypertension management after stroke and myocardial infarction: results from eclat1--a french nationwide study. Stroke.

[10] Brewer L, Mellon L, Hall P, Dolan E, Horgan F, Shelley E, Hickey A, Williams D (2015). Secondary prevention after ischaemic stroke: the aspire-s study. BMC Neurol.

[11] Heuschmann PUKJ, Nowe T, Dittrich R, Reiner Z, Cifkova R, Malojcic B, Mayer O, Bruthans J, Wloch-Kopec D, Prugger C (2015). Control of main risk factors after ischaemic stroke across europe: data from the stroke-specific module of the EUROASPIRE III survey. Eur J Prev Cardiol.

[12] Alvarez-Sabin JQM, Hernandez-Presa MA, Alvarez C, Chaves J, Ribo M (2009). Therapeutic interventions and success in risk factor control for secondary prevention of stroke. J Stroke Cerebrovas Dis..

[13] Jonsson AC, Hoglund P, Brizzi M, Pessah-Rasmussen H (2014). Secondary prevention and health promotion after stroke: can it be enhanced?. J Stroke Cerebrovas Dis.

[14] Lager KE, Mistri AK, Khunti K, Haunton VJ, Sett AK, Wilson AD. Interventions for improving modifiable risk factor control in the secondary prevention of stroke. The Cochrane database Syst Rev. 2014:Cd009103. 10.1002/14651858.CD009103.pub2 24789063

[15] Ihle-Hansen H, Thommessen B, Fagerland MW, Oksengard AR, Wyller TB, Engedal K, Fure B (2014). Multifactorial vascular risk factor intervention to prevent cognitive impairment after stroke and tia: a 12-month randomized controlled trial. Int J Stroke.

[16] Kronish IM, Goldfinger JZ, Negron R, Fei K, Tuhrim S, Arniella G, Horowitz CR (2014). Effect of peer education on stroke prevention: the prevent recurrence of all inner-city strokes through education randomized controlled trial. Stroke.

[17] McAlister FA, Majumdar SR, Padwal RS, Fradette M, Thompson A, Buck B, Dean A, Bakal JA, Tsuyuki R, Grover S (2014). Case management for blood pressure and lipid level control after minor stroke: prevention randomized controlled trial. CMAJ.

[18] O'Carroll RE, Chambers JA, Dennis M, Sudlow C, Johnston M (2014). Improving medication adherence in stroke survivors: mediators and moderators of treatment effects. Health Psychol.

[19] Glynn LG, Murphy AW, Smith SM, Schroeder K, Fahey T. Interventions used to improve control of blood pressure in patients with hypertension. Cochrane database Syst Rev. 2010:Cd005182. 10.1002/14651858.CD005182.pub4 PMC13248079 20238338

[20] Irewall ALÖJ, Bergström L, Laurell K, Söderström L, Mooe T (2015). Nurse-led, telephone-based, secondary preventive follow-up after stroke or transient ischemic attack improves blood pressure and ldl cholesterol: results from the first 12 months of the randomized, controlled nailed stroke risk factor trial. PLoS One.

[21] Joubert J, Reid C, Barton D, Cumming T, McLean A, Joubert L, Barlow J, Ames D, Davis S (2009). Integrated care improves risk-factor modification after stroke: initial results of the integrated care for the reduction of secondary stroke model. J Neurol Neurosurg Psychiatry.

[22] Kraft P, Hillmann S, Rucker V, Heuschmann PU (2017). Telemedical strategies for the improvement of secondary prevention in patients with cerebrovascular events-a systematic review and meta-analysis. Int J Stroke.

[23] Brotons C, Soriano N, Moral I, Rodrigo MP, Kloppe P, Rodriguez AI, Gonzalez ML, Arino D, Orozco D, Buitrago F (2011). Randomized clinical trial to assess the efficacy of a comprehensive programme of secondary prevention of cardiovascular disease in general practice: the preseap study. Revista espanola de cardiologia.

[24] Oral Abstracts. European Stroke Journal. 2017;2(IS)3–97 10.1177/2396987317705236

[25] Mooe TBL, Irewall A-L, Ögren J. The nailed stroke risk factor trial (nurse based age independent intervention to limit evolution of disease after stroke): Study protocol for a randomized controlled trial. Trials. 2013;14:5. 10.1186/1745-6215-14-5 PMC3551832 23289919

[26] Law MR, Morris JK, Wald NJ (2009). Use of blood pressure lowering drugs in the prevention of cardiovascular disease: meta-analysis of 147 randomised trials in the context of expectations from prospective epidemiological studies. BMJ.

[27] Snaterse M, Dobber J, Jepma P, Peters RJ, Ter Riet G, Boekholdt SM (2016). Effective components of nurse-coordinated care to prevent recurrent coronary events: a systematic review and meta-analysis. Heart.

[28] collaboration TR. Riksstroke annual report 2014; stroke and tia. 2015.

[29] Roumie CL, Zillich AJ, Bravata DM, Jaynes HA, Myers LJ, Yoder J, Cheng EM (2015). Hypertension treatment intensification among stroke survivors with uncontrolled blood pressure. Stroke.

[30] Group (2001). PC. Randomised trial of a perindopril-based blood-pressure-lowering regimen among 6,105 individuals with previous stroke or transient ischaemic attack. Lancet.

[31] Welin L, Bjalkefur K, Roland I (2010). Open, randomized pilot study after first stroke: a 3.5-year follow-up. Stroke.

[32] Irewall AL, Bergstrom L, Ogren J, Laurell K, Soderstrom L, Mooe T (2014). Implementation of telephone-based secondary preventive intervention after stroke and transient ischemic attack - participation rate, reasons for nonparticipation and one-year mortality. Cerebrovasc Dis extra.

