# Supplementary material for: Long-term, telephone-based follow-up after stroke and TIA improves risk factors: 36-month results from the randomized controlled NAILED stroke risk factor trial
Source: BMC Neurol. 2018 Sep 21;18:153. doi: 10.1186/s12883-018-1158-5 (PMC6148791; doi:10.1186/s12883-018-1158-5)
Supplement: Supplementary file 1 — Table S1. Baseline characteristics of the participants randomized in the trial. (DOCX 13 kb) [file 12883_2018_1158_MOESM1_ESM.docx]

**Supplementary table S1 -** Baseline characteristics of the participants randomized in the trial.

|  | Intervention (*n*=433) | Control (*n*=438) | *p* value |
| --- | --- | --- | --- |
| Mean age, years | 71.4 | 70.7 | ns |
| Women no. (%) | 191 (44.1) | 186 (42.5) | ns |
| *Qualifying even no. (%)t* |  |  |  |
| Ischemic stroke | 247 (57.0) | 261 (59.6) | ns |
| Intracerebral hematoma | 14 (3.2) | 17 (3.9) | ns |
| TIA | 172 (39.7) | 160 (36.5) | ns |
| mRS 3-5 no. (%) | 59 (13.6) | 52 (11.9) | ns |
| *Medical history no. (%)* |  |  |  |
| Stroke | 57 (13.2) | 50 (11.4) | ns |
| Myocardial infarction | 41 (9.5) | 40 (9.1) | ns |
| Heart failure | 21 (4.8) | 19 (4.3) | ns |
| Atrial fibrillation | 82 (19.4) | 79 (18.2) | ns |
| Diabetes | 67 (15.5) | 89 (20.4) | ns |
| Smoker no. (%) | 60 (13.9) | 63 (14.4) | ns |
| *Medication at 1 month no. (%)* |  |  |  |
| Antihypertensive drug | 308 (71.1) | 329 (75.5) | ns |
| Statin | 306 (74.5) | 334 (79.7) | ns |
| Antiplatelet drug | 327 (75.5) | 335 (76.5) | ns |
| Anticoagulant drug | 66 (16.1) | 65 (15.5) | ns |

*mRS* modified Rankin scale, *TIA* transient ischemic attack
